# Social Anxiety and Obsessive-Compulsive Disorder Are Common Among Persons With Multiple Sclerosis at King Abdulaziz Medical City, Riyadh

**DOI:** 10.7759/cureus.13619

**Published:** 2021-02-28

**Authors:** Ismail A Khatri, Sarah Aljwair, Hajar Alammar, Amjad Altariq, Nazish Masud, Yaser Al Malik, Suleiman Kojan

**Affiliations:** 1 Neurology, King Saud Bin Abdulaziz University for Health Sciences College of Medicine, Riyadh, SAU; 2 Neurology, King Abdullah International Medical Research Center, Riyadh, SAU; 3 Division of Neurology, Department of Medicine, King Abdulaziz Medical City, Ministry of National Guard Health Affairs, Riyadh, SAU; 4 Medical Education, King Saud Bin Abdulaziz University for Health Sciences, Riyadh, SAU; 5 Neurology, Oakland University William Beaumont School of Medicine, Royal Oak, USA

**Keywords:** multiple sclerosis, social anxiety disorder, obsessive compulsive disorder, saudi arabia, disability, psychosocial function

## Abstract

Background

Multiple sclerosis (MS) is associated with a physical disability and disturbed psychosocial functioning in young people. Many psychological and psychiatric comorbidities have been reported in MS.

Objective

To determine the frequency of social anxiety disorder (SAD) and obsessive-compulsive disorder (OCD) among MS patients and their relation to MS severity.

Methods

A cross-sectional survey was conducted in an adult MS cohort. Yale-Brown Obsessive-Compulsive Scale (Y-BOCS) and Social Phobia Inventory (SPIN) were used to determine the presence and severity of OCD and SAD. The Statistical Package for the Social Sciences (SPSS) version 22 (IBM Corp., Armonk, NY) was used for statistical analysis. The Mann-Whitney U test and logistic regression were used to assess the association of the two diseases with the severity of MS.

Results

A total of 145 persons with MS (pwMS) were studied. The mean age was 33.5 (±8.5) years; the mean duration of MS was 7.2 (± 5.1) years. The majority (74.1%) were women; 57.3% were married; 63% had a college education; 50% belonged to the higher middle-class socioeconomic strata. Relapsing-remitting multiple sclerosis was the most common type of MS (92.2%). The mean Expanded Disability Status Scale (EDSS) score was 2.24 (±2.19). SAD was reported by 26.9%, and OCD was reported by 31% of the cohort. PwMS with walking difficulty but not wheelchair-bound had a statistically significant increased risk of SAD (p = 0.036). There was no direct association between MS-related disability and OCD. However, pwMS with SAD were more likely to have concomitant OCD (t=4.68, p-value <0.001, 95% CI: 0.47-1.16). Increasing disability was associated with higher chances of developing social anxiety and, in turn, OCD (t=3.39, p-value <0.001, 95% CI: 0.66-2.52).

Conclusions

Social anxiety and obsessive-compulsive disorders were present in nearly one-third of pwMS. Impaired walking but not wheelchair dependence was associated with social anxiety. PwMS with SAD were more likely to have obsessive-compulsive disorder.

## Introduction

Multiple sclerosis (MS) is a neurodegenerative, progressive, multifocal demyelinating disease of the central nervous system [1]. The demyelination is caused by an immune-mediated inflammation likely resulting from an interaction between genetic and environmental factors [2]. The most common pattern of MS is the relapsing-remitting MS (RRMS) with a prevalence of (85%-90%), followed by secondary progressive MS (SPMS) and primary progressive MS (PPMS) [3]. The global prevalence of MS is 30 per 100,000, with an incidence rate of 2.5 per 100,000 per year [4]. Women constitute the majority of pwMS with a 2:1 ratio [4]. MS in the Middle East North Africa region has increased consistently with the growing global prevalence of the disease [5]. In a recent Saudi study, the prevalence of MS was 40.4/100,000 population, with three-fourths of pwMS younger than 40 years of age and a female to male ratio of 2:1 [6]. A local Saudi study from 1998 suggested that the onset was mostly in the third decade, with the majority of pwMS having RRMS [7].

A systemic review, investigating the prevalence of psychological disorders and their comorbidity in pwMS showed that the most common psychological comorbidities were depression and anxiety [8]. A Saudi study of 24 patients found that depression (20.8%) was more common than anxiety (4.2%) [9]. Prior studies have shown a high frequency of social anxiety disorder (SAD) and obsessive-compulsive disorder (OCD) among pwMS [10-11]. SAD is characterized by the fear of negative evaluation from others, somatic symptoms such as palpitation and sweating, social isolation, and avoidance behavior [10]. Epidemiological studies have shown the prevalence of SAD in the global population to be 2%-16% [12]. In a study of 251 pwMS, 30.6% had social anxiety symptoms [10]. OCD is characterized by a combination of obsessions such as repetitive thoughts and compulsions, i.e. repetitive behaviors [13]. Both are attributed to or exacerbated by anxiety. In the general population, OCD is estimated to have a 2% lifetime prevalence [12]. In a study of pwMS, OCD was found in 16.1% of pwMS that correlated with a higher EDSS score [11]. Previous studies evaluated the frequencies of SAD and OCD in pwMS in different cultural backgrounds [10-11]. One of the studies was performed in Canada, and the other was in the Iranian population. Both studies showed a significant burden of these conditions despite different cultural backgrounds.

SAD and OCD are among major causes of functional impairment worldwide [14-15]. They lead to social isolation and are associated with a low quality of life. Social anxiety is associated with occupational instability, poor economical and marital status [14]. OCD accounts for significant emotional and functional disability and severe family burden [15]. In pwMS, studying any aspect of the disease, including emotional and social aspects, which may affect the course of the disease or alter the disability is highly significant and may alter the management plan. There is very limited data on psychiatric comorbidities in pwMS from the region. Our study aimed to determine the frequency of social anxiety disorder and OCD among pwMS in a tertiary care center and to examine their association with the disability status of the patients.

This study was earlier presented as a poster at the 19th Department of Medicine Research Day at King Abdulaziz Medical City, Ministry of National Guard - Health Affairs (MNGHA), Riyadh, Saudi Arabia, on November 22, 2018.

## Materials and methods

Ethical approval for the study was obtained from the institutional review board (IRB) of King Abdullah International Medical Research Center (KAIMRC) and informed consent was obtained from persons with MS. A cross-sectional survey was conducted among pwMS visiting the outpatient neurology clinic between October 2017 and August 2018 at King Abdulaziz Medical City, MNGHA, Riyadh, Kingdom of Saudi Arabia. All adult pwMS, 16 years and above, who consented to participate were included. Multiple sclerosis was classified according to the clinical phenotype [16]. Those who did not have a conclusive diagnosis or had a cognitive disability were excluded from the study. A sample size of 139 was estimated with a margin of error of 5%, a confidence level of 95%, and a response distribution of 30.6% using the Raosoft sampling calculator (Raosoft, Inc., Seattle, WA) [10]. The non-probability convenience sampling technique was used for the inclusion of pwMS, as the data was collected in the hospital setting and pwMS were contacted based on availability at the time of data collection. A total of 145 pwMS were included in the study.

The Social Phobia Inventory (SPIN) and Yale-Brown Obsessive Compulsive Scale (Y-BOCS), both validated tools, were used to identify the presence of social anxiety and OCD symptoms [17-18]. The questionnaires were translated into Arabic by native Arabic speakers [19], and all the steps of translation, including forward and backward translation, were done followed by a pilot testing with 108 participants other than the ones included in the data analysis. The sample size of 108 for pilot testing was estimated based on the total number of items, which was 27, as recommended for reliability testing. Four pwMS per item were considered. Based on the pilot testing, item numbers 3, 6, 7, 8, 13, 14, and 15 of the SPIN questionnaire and item numbers 1, 2, 7, and 8 of the YOBCS questionnaire were reworded and the final questionnaire in Arabic was drafted. The Cronbach for the Y-BOCS 10 items was 0.90 while for the 17 items of the SPIN questionnaire, it was 0.91. The over-reliability score for the total 27 items of both questionnaires was 0.93, showing good internal reliability of the translated version of the questionnaires in our sample. The expanded disability status scale (EDSS) score was obtained from the treating neurologist’s assessment and was used for quantifying the disability in pwMS. Some patients suffered limitations, including visual impairment, motor disability, and illiteracy. For such pwMS, the questionnaire was filled by the co-investigators after asking the inventory questions in simple language and explaining the choice of answer.

The data were analyzed using the Statistical Package for Social Sciences (SPSS) version 22 (IBM Corp., Armonk, NY). The descriptive analysis was performed and the numerical variables were presented as mean and standard deviation, whereas the categorical data were presented as percentages and proportions. Initially, the total scores for SPIN and Y-BOCS were computed by a summation of all the items for each participant followed by the mean score. The EDSS score was categorized into fully ambulatory (EDSS ≤ 4.5), some walking difficulty (EDSS 5.0 - ≤ 6.5), and restricted to a wheelchair (EDSS ≥ 7) [20]. The cut-off level for having clinically significant symptoms of SPIN was ≥ 19 [17]. Additionally, SPIN and Y-BOCS were classified as mild, moderate, and severe based on the existing classification reported by the original developers of the questionnaire [17-18]. For measuring the association between SPIN categories and the EDSS, Fisher’s exact test was applied. Taking SAD as the grouping variable, and OCD and the EDSS score as the continuous variable, the Mann-Whitney U test was used to assess the relationship between the mentioned variables. Linear regression was applied to assess the predictors of social anxiety using the step-wise approach. The total SPIN score was taken as the dependent variable, whereas the Y-BOCS, age, duration of MS, and EDSS were included as predictors. After stepwise modeling, the age and duration of MS were removed from the model. For all the tests, a p-value of < 0.05 was considered significant.

## Results

A total of 145 pwMS participated in the study; 106 (73.1%) were females. The mean age of participants was 33.5± 8.5 years; the mean duration since the diagnosis of multiple sclerosis was 7.2±5.2 years. Ninety-two (63%) of the pwMS had a bachelor’s degree or higher, and 82 (57.3%) of all pwMS were married. Relapsing-remitting multiple sclerosis was the most common type of MS in 118 (92.2%) pwMS, followed by secondary progressive MS in five (3.9%) and primary progressive MS in three (2.3%). The demographic profile, type of multiple sclerosis, and current disease-modifying medication use are presented in Table 1.

**Table 1:** Demographic profile, type of multiple sclerosis, and current use of medications (n = 145)

Variables		Frequency	Percentage %
Age (Mean ±SD)		33.48±8.54	
Duration of MS± (Mean± SD)		7.16±5.15	
Gender	Female	106	73.1
	Male	37	25.5
	Missing information	2	1.4
Marital status	Single	56	39.2
	Married	82	57.3
	Divorced	5	3.5
Monthly family income	<10,000 riyals	38	27.9
	11,000 – 20,000 riyals	49	36.0
	>20,000 riyals	19	14.0
	Not known	29	21.3
Education	No education	4	2.8
	Elementary	5	3.4
	Middle	3	2.1
	High school	37	25.5
	Bachelor	85	58.6
	Master	6	4.1
	Doctor/ PhD	1	0.7
	Other	4	2.8
Type of MS	Relapsing Remitting MS	118	92.2
	Primary Progressive MS	3	2.3
	Secondary Progressive MS	5	3.9
	Unknown course	2	1.6
Medication	No medication	20	14.1
	Interferon beta 1a (Avonex)	18	12.7
	Interferon beta 1a (Rebif)	20	14.1
	Interferon beta 1b (Betaferon)	3	2.1
	Fingolimod (Gilenya)	32	22.5
	Teriflunomide (Aubagio)	8	5.6
	Dimethyl fumarate (Tecfidera)	2	1.4
	Natalizumab (Tysabri)	31	21.8
	Alemtuzumab (Lemtrada)	5	3.5
	Ocrelizumab (Ocrevus)	1	0.7
	Rituximab	2	1.4

Social anxiety disorder was reported by 39 (26.9%) of the participants, and OCD was reported by 45 (31%) of the participants. Most of the pwMS with SAD were categorized as mild 20 (51%), followed by moderate 13 (33%), and severe 5 (13%), while only one (3%) patient had extremely severe SAD. Among those who had OCD, mild symptoms 34 (76%) were the most prevalent, followed by moderate 9 (20%) and 2 (4%) had severe OCD, while none had extreme OCD. The EDSS score was available for 138 pwMS with a mean of 2.24 ± 2.19. Based on EDSS, 106 (77%) patients were fully ambulatory, 10 (7%) had some walking difficulty, and 2 (16%) were restricted to wheelchairs. The responses to various questions of SPIN and Y-BOCS are shown in Table 2. Disease severity and the proportions of SAD and OCD are presented in Table 3.

**Table 2:** Descriptive statistics of SPIN (1 – 17) and Y-BOCS (18 – 27) questions SPIN: Social Phobia Inventory; Y-BOCS: Yale-Brown Obsessive-Compulsive Scale

Descriptive statistics of SPIN and Y-BOCS					
	Not at all	A little bit	Somewhat	Very much	Extremely
	N (%)	N (%)	N (%)	N (%)	N (%)
1. I am afraid of people in authority	91 (64.1)	27 (19)	15 (10.6)	7 (4.9)	2 (1.4)
2.I am bothered by blushing in front of people	84 (58.3)	38 (26.4)	15 (10.4)	4 (2.8)	3 (2.1)
3. Parties and social events scare me	76 (52.8)	29 (20.1)	20 (13.9)	12 (8.3)	7 (4.9)
4. I avoid talking to people I don't know	80 (55.2)	34 (23.4)	18 (12.4)	9 (6.2)	4 (2.8)
5. Being criticized scares me a lot	58 (40)	48 (33.1)	15 (10.3)	16 (11)	8 (5.5)
6. Fear of embarrassment causes me to avoid doing things or speaking to people	75 (52.8)	35 (24.6)	20 (14.1)	6 (4.2)	6 (4.2)
7. Sweating in front of people causes me distress	96 (66.2)	24 (16.6)	13 (9)	9 (6.2)	3 (2.1)
8. I avoid going to parties	77 (53.1)	29 (20)	20 (13.8)	13 (9)	6 (4.1)
9. I avoid activities in which I am the center of attention	67 (46.2)	4 (29)	15 (10.3)	19 (13.1)	2 (1.4)
10. Talking to strangers scares me	102 (71.3)	24 (16.8)	13 (9.1)	2 (1.4)	2 (1.4)
11. I avoid having to give speeches	69 (47.6%)	32 (22.1)	19 (13.1)	17 (11.7)	8 (5.5)
12. I would do anything to avoid being criticized	71 (49.3%)	32 (22.2)	23 (16)	12 (8.3)	6 (4.2)
13. Heart palpitations bother me when I am around people	80 (55.9%)	33 (23.1)	20 (14)	6 (4.2)	4 (2.8)
14. I am afraid of doing things when people might be watching	64 (44.4%)	36 (25)	20 (13.9)	18 (12.5)	6 (4.2)
15. Being embarrassed or looking stupid is among my worst fears	60 (41.4%)	43 (29.7)	16 (11)	21 (14.5)	5 (3.4)
16. I avoid speaking to anyone in authority	89 (61.8%)	31 (21.5)	13 (9)	7 (4.9)	4 (2.8)
17. Trembling or shaking in front of others is distressing to me	67 (46.5%)	35 (24.3)	21(14.6)	10 (6.9)	11 (7.6)

**Table 3:** Frequency of social anxiety disorder, obsessive-compulsive disorder, and EDSS score among persons with MS EDSS: Expanded Disability Status Scale; SPIN: Social Phobia Inventory; Y-BOCS: Yale-Brown Obsessive Compulsive Scale

		n (%)
EDSS categories n = 138	Fully ambulatory	106 (77)
	Some walking difficulty	10 (7)
	Restricted to wheelchair	22 (16)
Severity of SAD based on SPIN score (n = 39)	Mild	20 (51)
	Moderate	13 (33)
	Severe	5 (13)
	Very Severe	1 (3)
Severity of OCD based on YOBCS score (n = 45)	Mild case	34 (76)
	Moderate case	9 (20)
	Severe case	2 (4)

Fisher’s exact test revealed a significant association between the EDSS scores, suggesting walking difficulty and clinically significant symptoms of social anxiety measured by SPIN with a p-value of 0.036. Social anxiety was more likely to be present in those pwMS who were not wheelchair-bound but walking with difficulty. There was a significant association of clinically significant SPIN (score≥19) with Y-BOCS using the two-tailed Mann-Whitney U test (U=1404; p-value <0.001). The Y-BOCS score of 145 pwMS had a mean of 6±6.02.

Multiple linear regression modeling showed Y-BOCS scores and EDSS together as a significant predictor of social anxiety (SPIN) with (t=4.68, p-value <0.001, 95% CI: 0.47-1.16) and (t=3.39, p-value <0.001, 95% CI: 0.66-2.52), respectively. Age and duration of MS were removed from the modeling based on the low R- squared value. The result of linear regression modeling suggested that pwMS who had social anxiety were more likely to have concomitant OCD and the higher the disability (EDSS scores), the higher the chance of social anxiety.

## Discussion

Social anxiety and OCD were frequent in our persons with multiple sclerosis. SAD was found in 39 (26.9%), which was significantly higher than the 2% - 16% frequency in the general population [12]. Studies have reported the prevalence of social anxiety 19% to 30% among pwMS [10,21]. A systematic review found the prevalence of social anxiety in pwMS to be 21.9% [8]. Our findings are consistent with a previously reported frequency of SAD in pwMS. The relative differences in the results can be partially attributed to the different scales used in the assessment of social anxiety. Interestingly, we found this relation most significant in those who were still ambulatory but had difficulty in walking. This has not previously been reported in the literature. In the study from Canada, although approximately 30% of pwMS reported anxiety, there was no correlation to neurological disability [10]. We believe that this finding can be the result of insecurity or fear of embarrassment of falling in public. We think this was due to the apprehension those pwMS felt while walking. Those who become wheelchair-bound probably accepted their disability more. Additionally, wheelchair-bound pwMS are probably less likely to attend social gatherings as compared to those who are still walking. One of the items in SPIN inquiring about anxiety related to trembling and shaking in public was reported to be extremely distressing by 11 (7.6%) and very much distressing by 10 (6.9%), again suggesting a possible feeling of embarrassment due to disability. Other questions that got the highest scores in SPIN were fear of being embarrassed, looking stupid, or being criticized, which are common features of social anxiety [10].

There are not many studies investigating the prevalence of OCD in Saudi Arabia in general and multiple sclerosis in particular. We found that 45 (31%) of the pwMS were suffering from OCD, which is much higher than the prevalence of OCD in the general population 2% [12]. Our findings were comparable with other studies that showed similar results in pwMS [22-24]. In one study, the prevalence of OCD among pwMS was somewhat lower at 16.1% [11]. These dissimilarities might result from the differences in sample size, cultural differences, time constraints, and educational levels among pwMS. In our study, obsessive thoughts were more common than compulsive behaviors.

Most of our pwMS had mild to moderate disability based on EDSS, hence the results may not be representative for pwMS with a severe disability. This could be attributed to the fact that most of the data were collected from outpatient clinics. Additionally, persons with severe MS are more likely to have cognitive dysfunction and were excluded from our study.

Our results also showed a significant relationship between elevated EDSS scores and social anxiety symptoms. Our results showed that pwMS with high EDSS scores were more likely to have SAD, and the pwMS who had SAD were, in turn, more likely to have OCD. Thus, the higher the EDSS scores the higher the chances of getting SAD as well as OCD. It was uncertain whether SAD developed first or OCD developed first. Social anxiety was probably more likely to develop first, as we found an independent relationship of SPIN scores to EDSS scores. Baldwin et al., exploring the relationship between OCD and SAD, thought that SAD was more likely to be secondary in pwMS with a primary OCD diagnosis [25].

There was no statistical significance between OCD or SAD and any of the other predictors, including age, gender, marital status, socioeconomic status, or the clinical subtype of MS, which was in concordance with other similar studies except that in Foroughipour et al., where women were more likely to get OCD [11]. One study suggested a correlation between OCD prevalence and higher educational level among people with MS [26]. We did not find any relationship between educational level and OCD.

The study's cross-sectional design limits establishing causal relationships. Other study limitations may include the use of self-administered questionnaires that might have led to over or under-reporting, which could not be ascertained. The study participants mostly had mild to moderate disability based on EDSS scores; in addition, all participants were recruited from a tertiary center.

Therefore, the results may not be generalizable for all MS populations. Our study assessed the psychiatric conditions based on self-reported questionnaires without formal psychiatric evaluation. A study with formal psychiatric assessment may address the limitation of self-administered questionnaires. Finally, it is essential to investigate other associated mental disorders and their burden on patients living with multiple sclerosis to remove any confounding bias.

## Conclusions

We found social anxiety and obsessive-compulsive disorder to be common among pwMS. There was an association between impaired walking and more prominent social anxiety symptoms. If SAD was present, the pwMS were more likely to have OCD, suggesting multiple mental comorbidities. These findings assert the need for psychiatric screening for persons with MS, as it is important to detect mental illnesses early for their large impact on pwMS lives and outcomes.
